# Dual-Responsive PIL Films with Gold Nanoparticles: Tailoring Electrical Responses to pH and Thermal Stimuli

**DOI:** 10.1021/acsami.4c14733

**Published:** 2024-10-23

**Authors:** Chia-Wei Chang, Sascha Benedict Lemich, Patrick Schütz, Siraphat Weerathaworn, Maria Weißpflog, Chia-Ti Wu, Yu-Hsuan Tseng, Chun-Ting Chang, Birgit Hankiewicz, Volker Abetz, Jiun-Tai Chen

**Affiliations:** † Department of Applied Chemistry, National Yang Ming Chiao Tung University, 300093 Hsinchu, Taiwan; ‡ Center for Emergent Functional Matter Science, National Yang Ming Chiao Tung University, 300093 Hsinchu, Taiwan; § Institute of Physical Chemistry, University of Hamburg, 20146 Hamburg, Germany; ∥ Institute of Membrane Research, Helmholtz-Zentrum Hereon, 21502 Geesthacht, Germany

**Keywords:** conductive, gold nanoparticles, PDMAEMA, pH-responsive, poly(ionic liquid), thermoresponsive, photo-RAFT

## Abstract

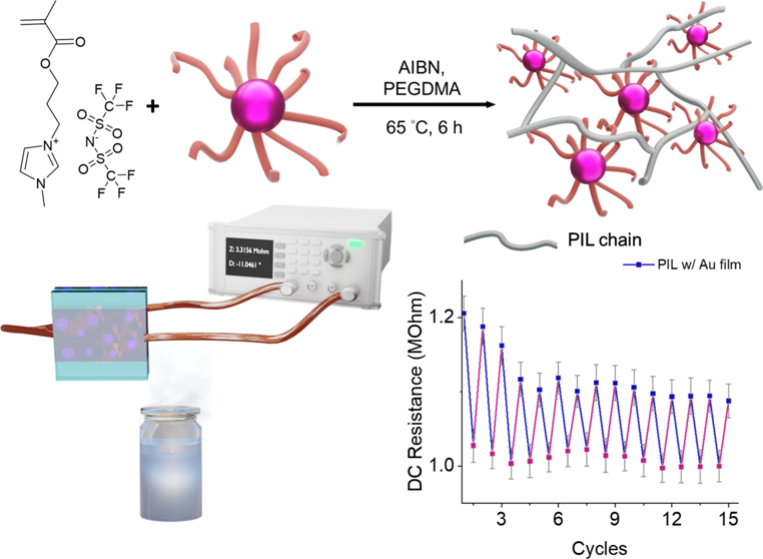

In recent years, stimuli-responsive poly(ionic liquids) (PILs) have attracted great attention. The stimuli-dependent properties, particularly the electrical properties, of multiresponsive PILs incorporating functionalized nanoparticles, however, have been less investigated. In this work, we present the synthesis, characterization, and application of PIL films incorporating pH- and thermoresponsive hybrid materials composed of gold nanoparticles functionalized with poly(2-(dimethylamino)ethyl methacrylate) (Au@PDMAEMA). The Au@PDMAEMA nanoparticles exhibit distinct responsiveness to changes in environmental pH and temperature, thereby altering the electrical properties of the PIL films blended with responsive gold nanoparticles (PIL w/Au). This research not only fills a gap in the study of electrical properties of multiresponsive nanoparticle-incorporated PILs but also extends the potential applications of PILs in various fields, including smart sensors and electronic devices.

## Introduction

In recent years, poly(ionic liquid)s (PILs) have drawn great attention because of their wide applications and unique properties, such as electronic devices,^1,2^ microextraction agents,^3−5^ bioengineering materials,^6,7^ and carbon dioxide absorption.^8−10^ To expand the applications of PILs, various researchers have developed PILs that are responsive to external stimuli, such as solvent, change in temperature, ionic strength, pH value, or light.^11−13^ PILs with responsive behaviors have been applied in various applications, such as surface-enhanced Raman scattering (SERS) substrates and separation for citrus flavonoids.^14,15^ Moreover, also multiresponsive PILs are reported.^16,17^

To achieve PILs with wider responsiveness, nanoparticle-incorporated multiresponsive PILs have been studied.^18−23^ For example, Tung et al. mixed PILs with silver nanoparticles and graphene oxide to fabricate chemo-resistive sensors of volatile organic compounds (VOCs).^24^ In another example, Teixeira et al. used PILs and gold nanoparticles to modify electrodes to detect the bactericide agent triclosan (TCS) in toothpaste and lake water using electrochemical cyclic voltammetry.^25^ Despite these works, stimuli-dependent properties, especially electrical properties, of multiresponsive PILs incorporating functionalized nanoparticles have been less investigated.

In this work, we have focused on the development and characterization of multiresponsive PIL films incorporating gold nanoparticles, aiming to explore and enhance their electrical properties under various stimuli. This endeavor addresses the existing gap in research regarding the responsive electrical properties of nanoparticle-incorporated PILs to different environmental conditions. Our approach involves the synthesis of PDMAEMA via photo-RAFT polymerization, ensuring precise control over its molecular weight and distribution. PDMAEMA is a well-known thermo- and pH-responsive polymer and is widely used as a drug delivery carrier and antibacterial material.^26−28^ This synthetic strategy facilitates the creation of the in situ synthesized and functionalized gold nanoparticles with specific responsiveness to temperature and pH variations,^29,30^ which are then integrated into PIL films.^31^ The innovative aspect of this work lies in the complex design of PIL blended with responsive gold nanoparticles (PIL w/Au) films, which demonstrates a unique dual responsiveness. The PIL with Au films are annealed by acidic and basic vapors to study their influence on conductivities.

Moreover, we have investigated the thermal and dynamic mechanical properties of the PIL w/Au films using techniques including differential scanning calorimetry (DSC), thermogravimetric analysis (TGA), and dynamic mechanical analysis (DMA). The results reveal improvements in the thermal stability and altered mechanical properties of the PIL films upon incorporation of Au@PDMAEMA nanoparticles. Furthermore, we have conducted studies on the electrical properties of the PIL w/Au films, observing enhancements in conductivity responsive to temperature and pH changes, which are characterized using methods such as Nyquist plots and Bode plots. As a result, by adding responsive Au nanoparticles, the electrical properties and stimuli responsiveness of the PIL films can be improved. This work not only contributes to the field of responsive materials by introducing a novel multiresponsive PIL system but also expands the potential applications of PILs under different environmental conditions.

## Results and Discussion

**Figure 1:**
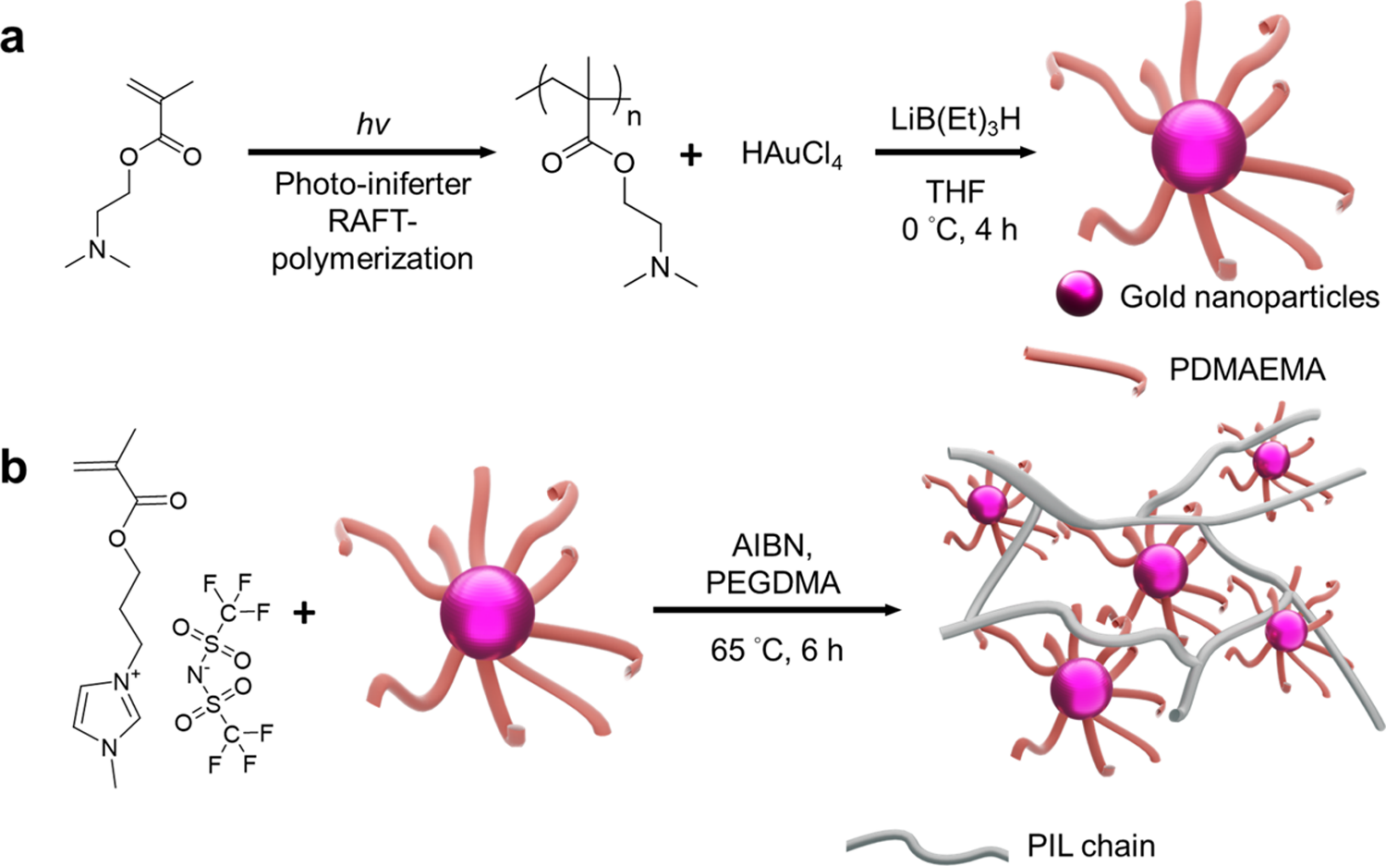
(a) Synthetic route of the responsive Au@PDMAEMA nanoparticles. (b) Fabrication of the PIL w/Au films.

The synthetic route of the responsive functionalized gold nanoparticles (Au@PDMAEMA) is illustrated in Figure 1a and described in the Experimental Section. In the first step, the PDMAEMA is synthesized by photoiniferter RAFT polymerization, as confirmed by the ^1^H-nuclear magnetic resonance (NMR) spectrum (Figure S1). Using poly(methyl methacrylate) calibrated size exclusion chromatography (SEC), the apparent weight-averaged molecular weight (*M*_w_) of the PDMAEMA is measured to be 12 kDa, as presented in Figure S2. In the second step, the functionalized Au@PDMAEMA nanoparticles are synthesized by an in situ approach.^31^ Therefore, the tetrachloroauric(III) acid trihydrate and the PDMAEMA are dissolved in THF. LiEt_3_BH in THF is then added and reduces the Au^3+^ ions to colloidal Au^0^ forming spherical gold nanoparticles, which were stabilized by the PDMAEMA simultaneously. The fabrication of the PIL films with the responsive Au@PDMAEMA nanoparticles (PIL with Au films) is shown in Figure 1b. First, the ionic liquid monomer 1-[(3-methacryloyloxy)propyl]-3-methylimidazolium bis(trifluoromethane) sulfonimide ([C3mim-MA][TFSI]), initiator 2,2′-azobis(2-methylpropionitrile) (AIBN), 10 wt % of cross-linker poly(ethylene glycol) dimethacrylate (PEGDMA), and 5 mL (∼7 mg or ∼0.105 wt % of gold determined by TGA) of Au@PDMAEMA nanohybrid colloid are evenly mixed and heated at 65 °C for 6 h. As a result, cross-linked PIL films encapsulating the responsive Au@PDMAEMA nanoparticles are formed. PDMAEMA is bound to the Au nanoparticles via the trithiocarbonyl group, which is therefore no longer available for radical polymerization during the PIL formation. The nanohybrids are mixed stably as the gold content is 10 times higher than reported in previous research.^32^ In this work, we check the transition characteristics of PDMAEMA by examining the electrical properties and surface wettabilities of the PIL w/Au films.

**Figure 2:**
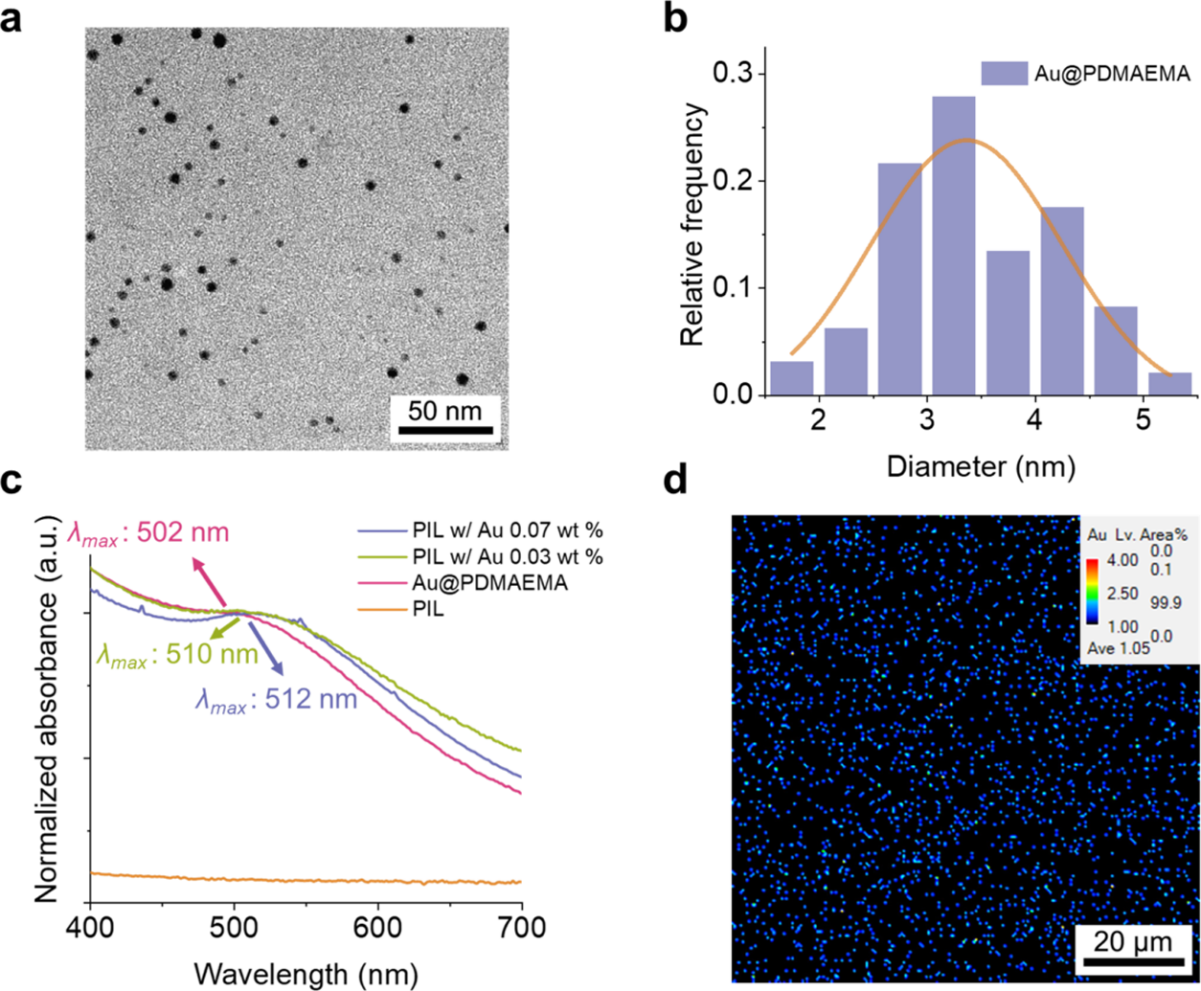
(a) TEM image of the responsive gold nanoparticles (Au@PDMAEMA). (b) Diameter distribution of responsive gold nanoparticles (Au@PDMAEMA). (c) UV–visible spectra of the pristine PIL and PIL w/Au films with 0.03 and 0.07 wt % of gold content and the colloidal responsive gold nanoparticles (Au@PDMAEMA) in dichloromethane. (d) Element distribution of gold within the PIL w/Au films by WDS mapping.

Figure 2a shows the transmission electron microscopy (TEM) image of the synthesized responsive Au@PDMAEMA nanoparticles. The responsive Au@PDMAEMA nanoparticles are distributed with less aggregation, and the corresponding size distribution observed by a Gaussian fit is presented in Figure 2b. Most of the nanoparticles are smaller than 5 nm, and the average diameter of the nanoparticles is 3.5 ± 0.8 nm. Figures 2c and S3 show the UV–vis spectra of the pristine PIL and PIL w/Au films (0.03 and 0.07 wt % of gold content) and the colloidal of the Au@PDMAEMA nanoparticles. The spectra of the solution of the Au@PDMAEMA nanoparticles in dichloromethane and the PIL w/Au films with 0.03 and 0.07 wt % of gold contents show broad absorption bands at ∼502, ∼510, and ∼512 nm, respectively, attributed to the absorption of the Au@PDMAEMA nanoparticles. It is reasonable to see the red-shifted peaks between the solution and film samples. The spectrum of the pristine PIL film, however, shows no absorption in the visible light regions. To determine the elemental distributions of gold in the PIL with Au films, an electron probe microanalyzer (EPMA) equipped with a wavelength dispersive spectrometer (WDS) is used. The WDS mapping is an essential and nondestructive instrument for analyzing material composition, and the technique enables the determination of the composition with an accuracy of up to 100 ppm.^33^ In Figure 2d, the WDS mapping confirms that the Au@PDMAEMA nanoparticles are blended in the cross-linked PIL films. Dark regions appear between regions with higher nanoparticle densities. So, the overall impression is that a network of gold particle chains lies beside dark regions without gold NPs, which may also enhance percolation and electrical conductivity. Energy-dispersive X-ray spectroscopy (EDS) and X-ray photoelectron spectroscopy (XPS) also confirm the compositions of the PIL w/Au films, as shown in Figures S4 and S5, respectively.

**Figure 3:**
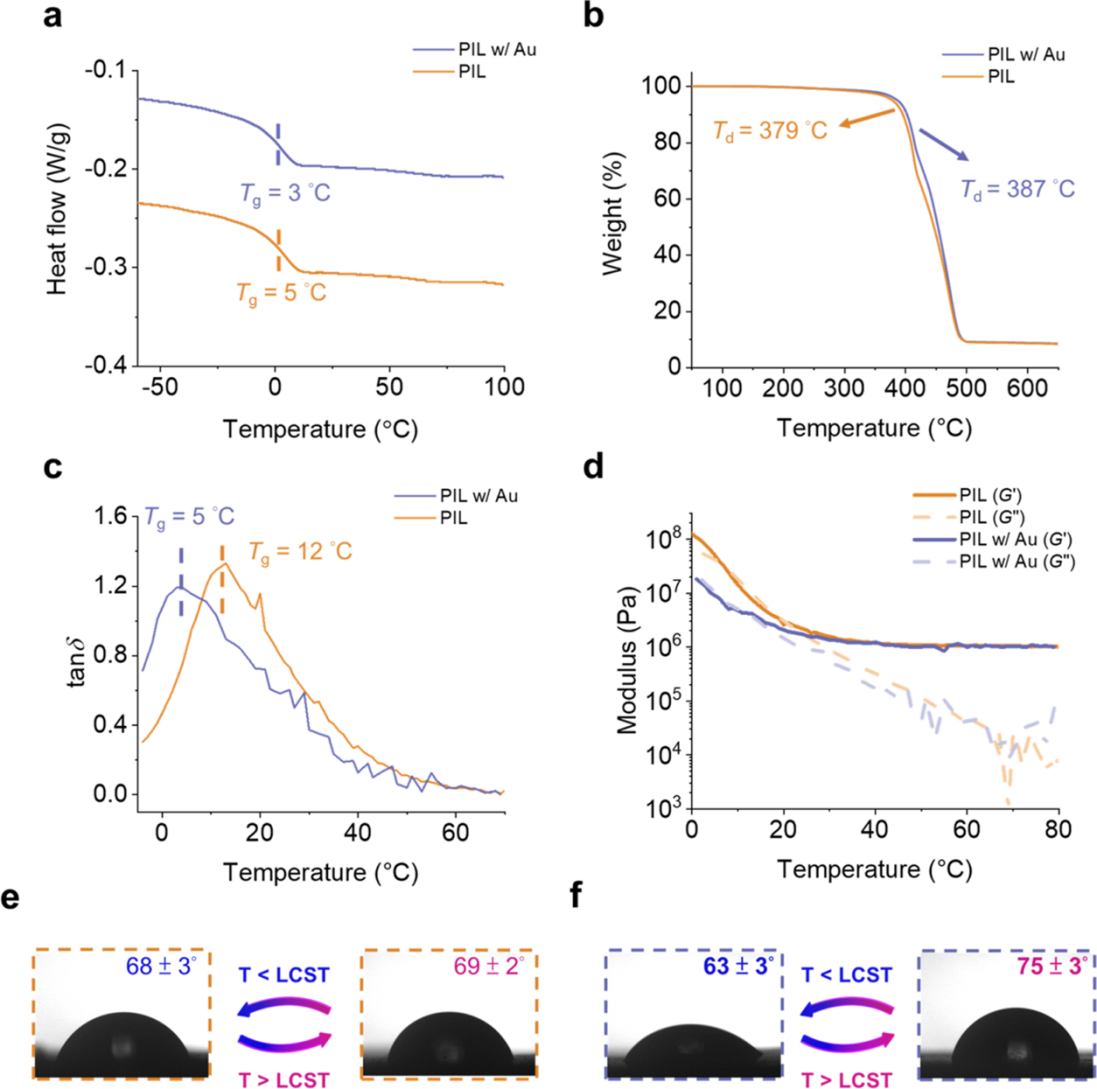
(a) DSC curve, (b) TGA curve, and (c, d) temperature-dependent DMA of the pristine PIL and PIL w/Au films. Water contact angles of the (e) pristine PIL and (f) PIL w/Au films below and above the LCST of PDMAEMA. The temperatures of the water contact angle measurements below and above the LCST are 25 and 60 °C, respectively.

To gain a deeper understanding of the thermal and mechanical properties of the pristine PIL and PIL w/Au films, differential scanning calorimetry (DSC), thermogravimetric analysis (TGA), and rheology measurements are also conducted. The DSC curves of the pristine PIL and PIL with Au films are presented in Figure 3a. The glass transition temperatures (*T*_g_) of the pristine PIL and PIL w/Au films under 10 K/min scanning rate are 5 and 3 °C, respectively. The glass transition temperatures (*T*_g_) of the pristine PIL and PIL w/Au films under different scanning rates (10, 5, and 2.5 K/min) are presented in Figure S6 and Table S1. The *T*_g_s of the PIL films slightly decrease after mixing the Au@PDMAEMA nanoparticles at different scanning rates. Also, the *T*_g_s of the pristine PIL and PIL w/Au films decrease under lower scanning rates. The cloud point temperature (*T*_c_), indicating the LCST of the PDMAEMA, can also be seen at 42–43 °C for the aqueous solution of 1 wt % PDMAEMA, as shown in Figure S7. The thermal stability of the pristine PIL and PIL w/Au films are measured by TGA (Figure 3b). The degradation temperatures (*T*_d_) at 5 wt % loss of the pristine PIL and PIL w/Au films are 379 and 387 °C, respectively, demonstrating that the thermal stability increases after doping the Au@PDMAEMA nanoparticles in the PIL films. Figure 3c presents the DMA curves of the pristine PIL and PIL w/Au films, in which the loss factor (tan δ), defined by the ratio of the loss modulus (*G*″) and storage modulus (*G*′), at different temperatures can be observed. The dynamic glass transition temperature *T*_g_ determined at the frequency of 10 rad/s of the pristine PIL and PIL w/Au films can also be obtained from the maximum values of tan δ to be 12 and 5 °C, respectively. Although the determined values of *T*_g_ depend on the method used (here: DSC and DMA), the same trend is observed. The chain motions are influenced by the Au@PDMAEMA nanoparticles, which further alter the thermomechanical properties of the PIL w/Au films.^32^ DMA was used to investigate the viscoelastic behavior of the materials. First of all, the amplitude sweep curves were recorded to select the suitable amplitude in temperature-dependent DMA, which should be in the linear viscoelastic region (LVER) as displayed in Figure S8.

Figure 3d presents the temperature-dependent curves of loss modulus (*G*″) and storage modulus (*G*′) of the PIL w/Au films. The storage moduli in the range of ∼20–60 °C indicate the rubbery plateau region, confirming the cross-linked network of the PIL w/Au films. According to the literature, the lower critical solution temperatures (LCST) of PDMAEMA are affected by molecular weights, pH values, and concentrations of electrolytes in the solutions.^34−36^ In general, the higher the degree of polymerization, the lower the LCST. For Au@PDMAEMA_39_ particles and Au@PDMAEMA_92_ particles, the LCST are 46–51 and 38–42 °C, respectively.^31^ Because the apparent weight-averaged molecular weight (*M*_w_) of the PDMAEMA synthesized in this work is measured to be 12 kDa, which corresponds to Au@PDMAEMA_77_, the LCST of the particles is estimated to be 42–45 °C. The water contact angles of the pristine PIL and PIL w/Au films below and above the LCST of PDMAEMA are shown in Figure 3e,f, respectively. For the pristine PIL films, the values of the water contact angles show no obvious differences below and above the LCST of PDMAEMA. By comparison, the values of the water contact angles of the PIL w/Au films exhibit more apparent changes from 63 ± 3 to 75 ± 3° when the films are heated above the LCST of PDMAEMA. The results demonstrate the effect of the Au@PDMAEMA on the surface hydrophilicities of the PIL films above the LCST. The temperatures of the water contact angle measurements below and above the LCST are 25 and 60 °C, respectively. The values of the *T*_g_, *G*′, and *T*_d_ of the pristine PIL and PIL w/Au films are summarized in Table 1.

**Table 1:** Summary of the Glass Transition Temperatures (*T*_g_), Storage Modulus (*G*′), and Temperature of Degradation at 5% (*T*_d_) of the Pristine PIL and PIL w/Au Films

	*T*_g, DMA_ (°C)	*T*_g, DSC_ (°C)	*G*′_0 °C_ (MPa)	*G*′_70 °C_ (MPa)	*T*_d_ (°C)
PIL	12	5	126	1.06	379
PIL w/Au	5	3	108	1.04	387

**Figure 4:**
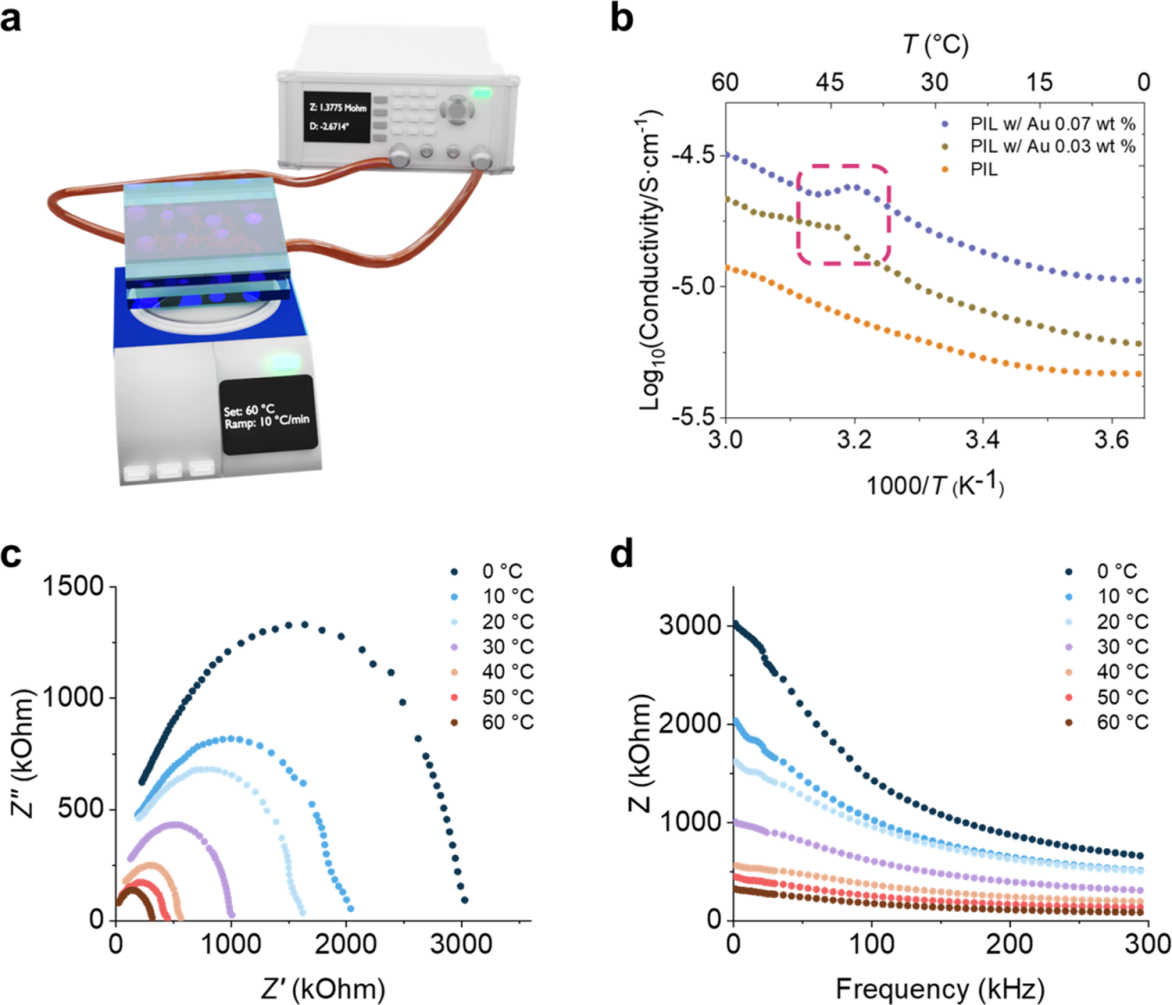
(a) Schematic illustration of the setup to investigate the temperature-responsive change of the electrical properties of the PIL w/Au films. (b) Temperature-dependent conductivity plot of the pristine PIL, PIL w/Au films with 0.03 and 0.07 wt % of gold content. (c) Nyquist plots and (d) Bode plots of the PIL w/Au films at different temperatures.

Furthermore, we investigate the temperature and pH-responsive electrical properties of the pristine and PIL w/Au films. Figure 4a illustrates the measurements of the DC resistances, Nyquist plots, and Bode plots of the PIL w/Au films under different temperatures. The LCR (inductance (*L*), capacitance (*C*), and resistance (*R*)) meter is used to obtain the data, and a hot–cold plate is used to control the temperature precisely. Figure 4b shows the temperature-dependent conductivity plots. After the Au@PDMAEMA nanoparticles are mixed into the PIL films, the conductivities increase by ∼100% because of the conductivities of the nanoparticles. The Nyquist plots and Bode plots of the pristine PIL and PIL w/Au films are shown in Figure S9. More importantly, the conductivities of the PIL w/Au films are partially inhibited at ∼40 °C, owing to the transition of the PDMAEMA chains. With different loading levels of responsive gold nanoparticles (0.03 and 0.07 wt % gold content), the conductivities of the PIL w/Au films present a similar trend at ∼40 °C. Therefore, the conductivities of the films increase when the temperatures increase because the charge carrier mobilities increase;^37^ the temporary decrease in conductivities is mainly contributed by the transition of the PDMAEMA chains. For PIL films with responsive gold nanoparticles (0.03 and 0.07 wt %), it can be seen that the conductivities and slopes of the curves are different above and below the transition temperatures of the responsive gold nanoparticles. The temperature-dependent conductivity plots and the linear fit results are presented in Figure S10. The differences between the fit and measured conductivities are summarized in Table S3, presenting the thermal-responsive electrical properties of the PIL with Au films with different loading levels. The transition regions are highlighted by a dashed square. Figure 4c,d presents the Nyquist and Bode plots of the PIL w/Au films, respectively. At higher temperatures, the x-intercepts of the Nyquist plots and the *y*-intercepts of the Bode plots all become smaller, indicating the higher conductivities of the PIL w/Au films. Previously, Cowan et al. reported curable cross-linked PIL copolymer films with conductivities of ∼10^–8^ S·cm^–1^ at 25 °C.^38^ Also, O′Harra et al. developed cross-linked PIL films with conductivities of ∼1.7 × 10^–8^ S·cm^–1^ at 30 °C.^39^ In this work, the conductivities of the cross-linked PIL films are ∼6.0 × 10^–6^ S·cm^–1^ at 25 °C, much higher than those reported by Cowan et al. and O′Harra et al., which might be due to the types and amounts of monomers and cross-linkers. After doping the Au@PDMAEMA nanoparticles into the PIL films, the conductivities of the PIL with Au films are further increased to 1.5 × 10^–5^ S·cm^–1^ at 25 °C.

**Figure 5:**
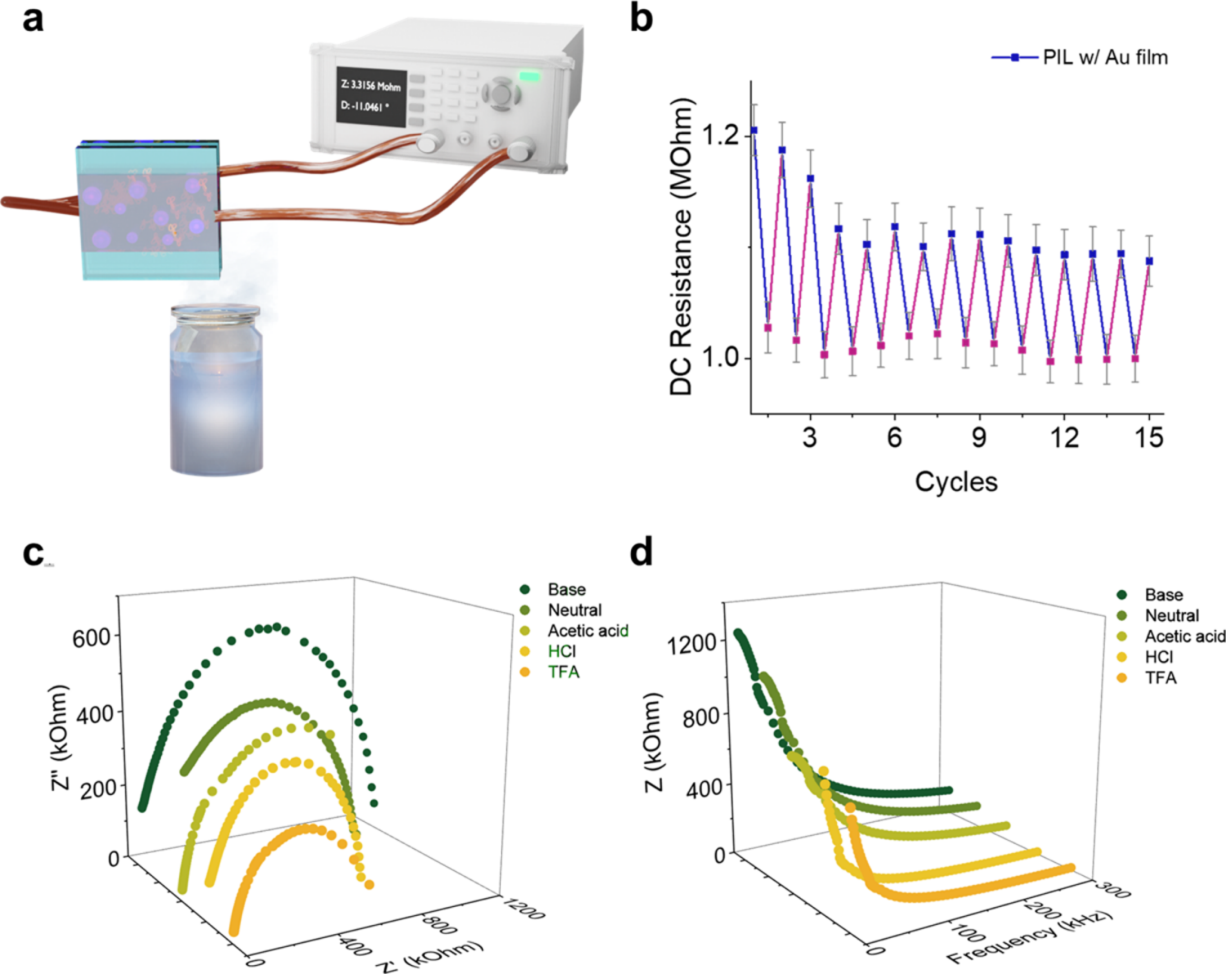
(a) Schematic illustration of measuring the pH-responsive electrical properties of the PIL w/Au films with 0.07 wt % of gold content. Reversible electrical properties of (b) DC resistance, three-dimensional (c) Nyquist plots, and (d) Bode plots of the PIL w/Au films under different pH values.

Figure 5a demonstrates the measurement device of the pH- and temperature-dependent electrical properties of the PIL w/Au films. When the films are annealed by acidic and basic vapors, the films have different electrical properties. The reversible pH-dependent electrical properties of the PIL w/Au films are shown in Figure 5b–d. Figure 5b presents the values of the DC resistance of the PIL with Au films. When the films are annealed by acidic vapors (hydrochloric acid, p*K*_a_ = 2), the polymer side chains of the Au@PDMAEMA hybrid materials are protonated and positively charged; therefore, the DC resistances decrease. The conductivities of the PDMAEMA chains increase when the chains are protonated and form the PDMAEMA-Cl salt in the bulk state.^40^ Therefore, when the PIL w/Au films are annealed by acidic vapors, the conductivities of the films increase. On the contrary, when the films are annealed by alkaline vapors (ammonium hydroxide), the polymer chains are deprotonated and discharged, causing an increase in the DC resistances. Under 15 cycles of acid (hydrochloric acid) and alkaline (ammonium hydroxide, p*K*_b_ = 4.75) vapor annealing, the PIL w/Au films exhibit pH responsiveness; the resistance change of each cycle is 10 ± 2%. The resistance changes under other types of acidic vapor annealing while keeping the same alkaline (ammonium hydroxide) vapors annealing are shown in Figure S11. As summarized in Table S3, the resistance changes of each cycle under trifluoroacetic acid (p*K*_a_ = 0.74), hydrochloric acid (p*K*_a_ = 2), and acetic acid (p*K*_a_ = 4.76) are 12 ± 3, 10 ± 2, and 5 ± 1%, respectively. As expected, under acidic vapors annealing with higher p*K*_a_ values, a lower level of protonation of PDMAEMA chains is induced, resulting in smaller resistance changes of the PIL with Au films. In general, PIL films can be used as absorbing materials, especially for organic solvents and acids.^41−43^ Therefore, under acid vapor annealing, the PIL films can absorb the acid vapor which protonates the PDMAEMA. When the samples are then exposed to alkaline vapors, the protonated PDMAEMA may transfer the proton to ammonia, which will then stay as NH_4_Cl salt in the film as it cannot evaporate. not be fully neutralized; as a result, the resistance values are not fully reversible, and a drift is observed. Figure 5c,d shows the Nyquist and Bode plots of the PIL w/Au films under different pH values. The results also demonstrate that the higher conductivities of the PIL w/Au films can be achieved under acidic vapors while the lower conductivities are attained under basic vapors. In this work, the pH-responsive electrical properties are conducted at 25 °C. Previously, it has been reported that PDMAEMA chains present pH responsiveness even above LCST.^34^ The top-view and cross-sectional view SEM images of the PIL w/Au films with 0.07 wt % of gold content before and after acid and alkaline vapors annealing are shown in Figure S12, where the film thicknesses remain ∼950 μm before and after over 50 cycles of acid and alkaline vapors annealing.

## Conclusions

In conclusion, we successfully synthesized and characterized responsive functionalized gold nanoparticles (Au@PDMAEMA). PDMAEMA was obtained by photo-RAFT polymerization. Taking advantage of the sulfur-containing photoiniferter RAFT agent, the polymers could be grafted onto the gold nanoparticles. Au@PDMAEMA could be incorporated into the PIL during its synthesis by free radical polymerization. Notably, these hybrid materials exhibit distinct pH- and thermoresponsive properties, as demonstrated by the changes in conductivity under varying environmental conditions. Integrating Au@PDMAEMA nanoparticles into the PIL films enhances their thermal stability and alters their thermomechanical properties, as evidenced by the DSC, TGA, and DMA. These modifications are further reflected in the altered glass transition temperatures (*T*_g_) and storage moduli (*G*′) of the films. The electrical properties of the PIL w/Au films, particularly their conductivity, are influenced by temperature and pH, showcasing their potential in applications requiring responsive materials. The reversible acid–base electrical properties further underline the versatility and adaptability of these films. This study not only advances our understanding of the behavior of responsive nanoparticle-incorporated PIL films but also opens avenues for their applications in various fields requiring materials with adjustable physical and chemical properties.

## Experimental Section

### Materials

2-(Dimethylamino)ethyl methacrylate (DMAEMA, 98%), gold(III) chloride trihydrate (HAuCl_4_·3H_2_O, 99.9%), 1.0 M lithium triethylborohydride in THF (Super-Hydride), 1-methylimidazole (ReagentPlus, 99%), 3-bromo-1-propanol (97%), poly(ethylene glycol) dimethacrylate (PEGDMA, *M*_n_ ∼ 550 g/mol), lithium bis(trifluoromethanesulfonimide) (LiTFSI, 99%), 4-methoxyphenol (ReagentPlus, 99%), 2,2′-azobis(2-methylpropionitrile) (AIBN, 98%), methacryloyl chloride (97%), and acetonitrile (anhydrous, 99.8%) were acquired from Sigma-Aldrich. Tetrahydrofuran (THF, extra dry with molecular sieve, 99.7%) and dimethylformamide (DMF, 99.9%) were obtained from VMR chemicals. Triethylamine, dichloromethane (DCM, anhydrous, 99.8%), and ethanol were purchased from Echo Chemical. 1,4-Dioxane (99%) and cyclohexane (99.5%) were obtained by Grüssing. 4-Cyano-4-(dodecyl sulfanyl thiocarbonyl)sulfanyl pentanoic acid (97%, CDTPA) was obtained by ABCR. Trifluoroacetic acid (TFA, 99%) was purchased from Thermo Fisher Scientific. Acetic acid (99.8%) was obtained from Honeywell Fluka.

### Preparation of Responsive Gold Nanoparticles (Au@PDMAEMA)

Responsive gold nanoparticles (Au@PDMAEMA) were prepared according to the previous literature.^31,44^ 2-(Dimethylamino)ethyl methacrylate (DMAEMA) and 1,4-dioxane were filtrated by a basic aluminum oxide column to remove the inhibitor. 2.5 g of DMAEMA and 50 mg of RAFT agent 4-cyano-4-(dodecyl sulfanyl thiocarbonyl)sulfanyl pentanoic acid (CDTPA) were dissolved in 10 mL of 1,4-dioxane, and 100 μL of DMF was also added to the solution as the ^1^H NMR reference. The mixture was purged by nitrogen for 15 min. The reaction was under a nitrogen atmosphere and illuminated by green light (513 nm, 0.60 mW cm^–2^) at 70 °C for 5 h. The product was precipitated by cyclohexane three times to remove unreacted monomer and RAFT agent. After precipitation, the polymer was dissolved in THF and dried in vacuum at room temperature.

28 mg of gold(III) chloride trihydrate (69 μmol) was dissolved in 20 mL of anhydrous THF and degassed and dried in a three-neck bottle. A solution of 83 mg of PDMAEMA (*M*_w_ = 12 kDa, 6.9 μmol) in 20 mL of anhydrous THF was added dropwise to the gold solution over 10 min. The reaction was kept under nitrogen flow, cooled to 0 °C, and kept under these conditions throughout the reaction for 15 min. Then, 0.45 mL of Super-Hydride was added to the light-yellow solution, and the mixture was vigorously stirred for 4 h. The dark-purple solution was precipitated by cyclohexane, and the precipitate was isolated by centrifugation (3000 G, 5 min), redissolved by DCM, and filtrated. The residue was dissolved in DCM (30 mL), sonicated for 10 min, and stored at 4 °C.

### Preparation of PIL w/Au Films

The synthetic routes of ionic liquid monomer [C_3_mim-MA][TFSI] were shown in the previous research.^45,46^ The PIL w/Au films were fabricated by mixing 1 g (1.85 mmol) of ionic liquid monomer [C_3_mim-MA][TFSI], 10 mg (0.06 mmol) of initiator azobis(isobutyronitrile) (AIBN), 0.1 g of cross-linker poly(ethylene glycol) dimethacrylate (PEGDMA, *M*_n_ ∼ 550 g/mol), and or 5 mL (∼7 mg or ∼0.14 wt % of gold determined by TGA) of the responsive gold nanoparticles (Au@PDMAEMA) solution in DCM. The composite solution was subsequently introduced into the gap between two glass substrates that had been surface-modified with chlorosilanes. The Teflon films were used to control the thicknesses of the PIL w/Au films, which were 1 mm. The prepared sample was subsequently placed in an oven with a consistent temperature of 65 °C for 6 h.

### Electrical Property Measurements

The temperature- and pH-dependent conductivities of the pristine PIL and PIL w/Au films were measured by an LCR meter (C_p_-R_p_ mode) with a frequency of 1 kHz and a voltage of 1 V. The temperature gradient of the hot/cold plate was set at 10 °C/min, from 0 to 60 °C. The Nyquist and Bode plots were recorded in Z-θ_d_ mode. The frequencies were from 0.01 to 300 kHz, and the voltage was set at 1 V. The acidic vapors were generated by trifluoroacetic acid (TFA, p*K*_a_ = 0.74), 0.01 M HCl (p*K*_a_ = 2), and acetic acid (p*K*_a_ = 4.76), and the alkaline vapors were produced by 1 M NH_3_ (p*K*_b_ = 4.75) at 298 K in a glass chamber with a volume of 1.5 L, respectively. After 30 min, the samples were placed into the chamber for 1 h. An LCR meter (GW Instek, LCR-6300) was used to measure the thermal and pH-responsive electrical properties of the PIL w/Au films with 0.07 wt % of gold content.

### Structure Analysis and Characterization

A transmission electron microscope (TEM, JEOL JEM-1011) using an acceleration voltage of 100 kV was used to obtain the size and distribution of Au@PDMAEMA nanoparticles. 100 particles were manually measured using the software ImageJ (ver. 1.53) to obtain the size distribution curves with a nonlinear curve (Gaussian) fit. Data evaluation and linear fit of the temperature-dependent conductivity curves were performed by OriginPro 2018 64-bit (ver. 9.5, OriginLab Cooperation). A Modular Compact Rheometer (MCR 502, Anton Paar GmbH) was used to measure the storage moduli and loss moduli as well as dynamic glass transition temperature *T*_g_ of the pristine PIL and PIL w/Au films with ω = 10 rad/s and γ = 0.001%. The gap between a Peltier plate and an 8 mm plate–plate geometry was set at 1 mm covered by a chamber with controlled heating and a constant nitrogen flow. To confirm that selected strain amplitude was in the linear viscoelastic regime (LVER), amplitude sweep was conducted from 0.001 to 10% with ω = 10 rad/s and temperature 25 °C. Temperature-dependent measurements were done with the temperature ranging from 70 to −5 °C and with rates of 1 K min^–1^. All of the data were then interpreted by RheoCompass software (version 1.19.266). Size exclusion chromatography (SEC) with a Polymer Standard Service Gram column and a VWR LaChrom L-2490 refractive index detector was used to obtain the apparent weight-average molecular weight (*M*_w_) using narrowly distributed poly(methyl methacrylate) standards. The mobile phase was *N*,*N*-dimethylacetamide with 0.1 M LiCl and a flow rate set at 0.1 mL/min. The experiment was measured at 30 °C. A hot/cold plate (Model 35150, Ugo Basile) was used to control the temperature. The cloud point of PDMAEMA was observed by heating the PDMAEMA solution. The 1 wt % aqueous solution of PDMAEMA was placed on the hot/cold plate at 35 °C for 10 min and heated to 55 °C at a rate of 1 °C/min. A First Ten Angstrom (FTA125) contact angle meter, fitted with a CCD camera, was employed to determine the water contact angles of the samples. The PIL w/Au films were measured under 25 and 60 °C to test the thermal-responsive properties of PDMAEMA. The top-view and cross-sectional view images of the pristine PIL and PIL w/Au films are obtained from a scanning electron microscope (SEM, JEOL JSM-7401F) with an acceleration voltage of 5 kV. An energy-dispersive X-ray spectrometer (EDS, Oxford EDS 7585) was used to measure the elemental compositions of pristine PIL and PIL w/Au films. An X-ray photoelectron spectrometer (XPS) and a high-resolution X-ray photoelectron spectrometer (ULVAC-PHI, PHI Quantera II) with a scanning X-ray microprobe (Al anode), a 180° hemispherical analyzer, and a 128-channel detector were also used. A differential scanning calorimeter (DSC) 204 F1 Phoenix (NETZSCH Gerätbau GmbH) was used to determine thermal properties. 5–10 mg of samples were weighed on an aluminum crucible under nitrogen atmosphere. The thermograms were recorded covering temperatures between −90 and 110 °C at a 10 K min^–1^ heating rate. Data were then processed by Proteus analysis (NETZSCH Gerätebau GmbH). The glass transition temperatures (*T*_g_) of the pristine PIL and PIL w/Au films under different scanning rates were measured by a differential scanning calorimeter (DSC) (DSC 250, TA Instruments) and processed by the TRIOS analysis software (TA Instruments). The thermograms were recorded covering temperatures between −80 and 120 °C with 10, 5, and 2.5 K min^–1^ heating rates. The second heating round spectra are shown in the figure. A thermogravimetric analyzer (TGA) (TGA55, TA Instruments) was used to measure the difference in thermal stability of the pristine PIL and PIL w/Au films. The samples were heated from 50 to 700 °C at a speed of 20 °C/min under a nitrogen-purged (60 mL/min) atmosphere. UV–vis extinction spectra of the Au@PDMAEMA solution were recorded using a UV5 spectrophotometer (Mettler Toledo) at 20 °C. The solution was diluted by DCM and measured in a quartz cuvette with a concentration of ∼0.07 wt % gold and an optical length of 1 cm. The films were measured in a self-built measurement cell to allow the laser to penetrate the film at a specific location (one measurement point). ^1^H NMR measurements were performed with a Bruker Avance II 400 MHz instrument at 300 K with CDCl_3_ as the solvent. During polymerization, an internal DMF standard was used to track the monomer conversion. Data processing was performed using MestReNova (ver. 12.0.0, Mestrelab Research S.L.9). The WDS elemental mapping was obtained by a High-Resolution Hyper Probe (JXA-iHP200F, JEOL). The acceleration voltage was 12 kV, and the scanning range was 256 × 256 μm^2^.

## Supporting Information Available

1H NMR and SEC spectra of PDMAEMA synthesized by photoiniferter RAFT polymerization; EDS and XPS spectra of the PIL w/Au films; DSC spectra of the pristine PIL and PIL w/Au films under different scanning rates; SEM images of the pristine PIL and PIL w/Au films before and after acid and base vapors annealing; and pH-responsive electrical properties of the PIL w/Au films (PDF)
