# Supplementary material for: Dual-Responsive PIL Films with Gold Nanoparticles: Tailoring Electrical Responses to pH and Thermal Stimuli
Source: ACS Appl Mater Interfaces. 2024 Oct 23;16(44):61096–104. doi: 10.1021/acsami.4c14733 (PMC11551959; doi:10.1021/acsami.4c14733)
Supplement: Supplementary file 1 — am4c14733_si_001.pdf [file am4c14733_si_001.pdf]

Supporting Information

Dual-Responsive PIL Films with Gold Nanoparticles: Tailoring Electrical Responses to pH and Thermal Stimuli

Chia-Wei Chang,[1] Sascha Benedict Lemich,[3] Patrick Schütz,[3] Siraphat Weerathaworn,[3] Maria Weißpflog,[3] Chia-Ti Wu,[1] Yu-Hsuan Tseng,[1] Chun-Ting Chang,[1] Birgit Hankiewicz,[3] Volker Abetz,[3,4]* and Jiun-Tai Chen[1,2]*

[1]Department of Applied Chemistry, National Yang Ming Chiao Tung University, 300093 Hsinchu, Taiwan

[2]Center for Emergent Functional Matter Science, National Yang Ming Chiao Tung University, 300093 Hsinchu, Taiwan

[3]Institute of Physical Chemistry, University of Hamburg, 20146 Hamburg, Germany

[4]Institute of Membrane Research, Helmholtz-Zentrum Hereon, 21502 Geesthacht, Germany

*To whom correspondence should be addressed. E-mail: volker.abetz@hereon.de (V. Abetz), and jtchen@nycu.edu.tw (J.T. Chen).

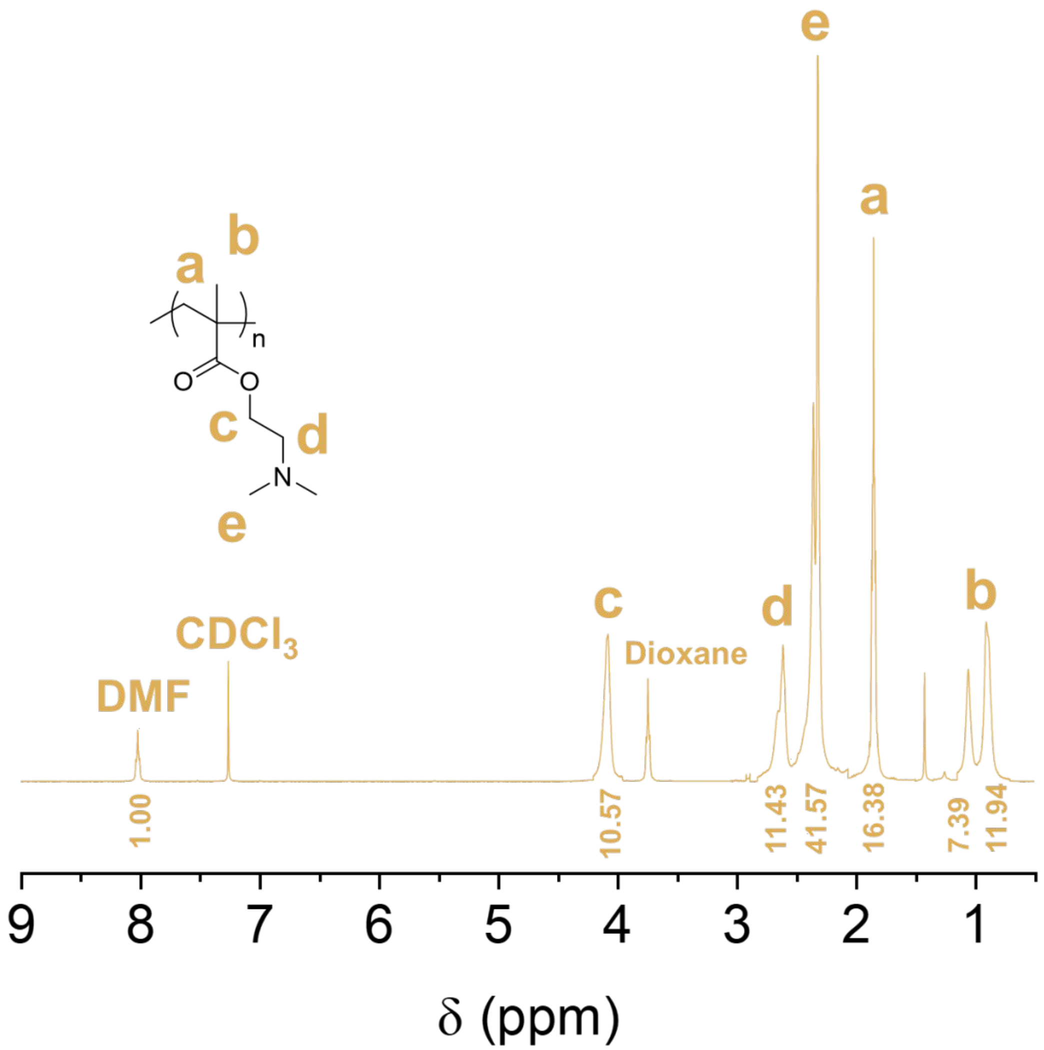

Figure S1. ^{1}H-NMR spectrum of PDMAEMA.

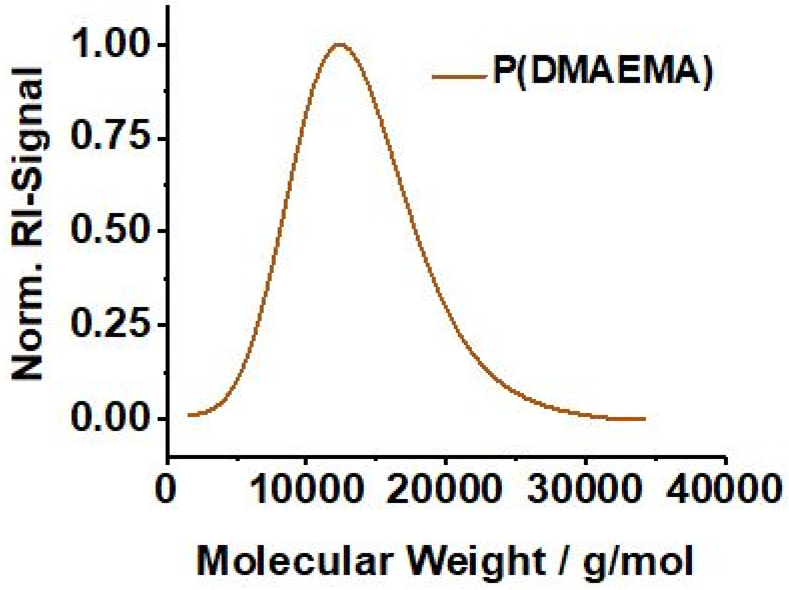

Figure S2. SEC of PDMAEMA, calibrated with PMMA-standards.

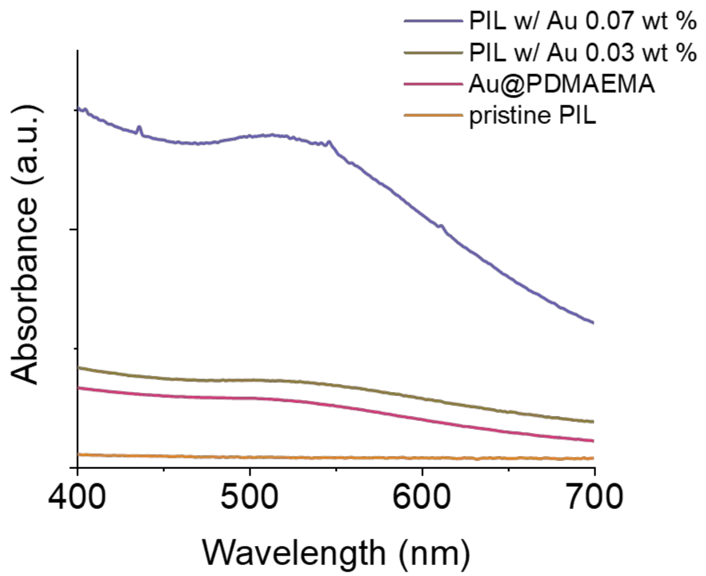

Figure S3. UV-visible spectra of the pristine PIL and PIL w/ Au films with 0.03 and 0.07 wt % of gold content and the colloidal responsive gold nanoparticles (Au@PDMAEMA) in dichloromethane.

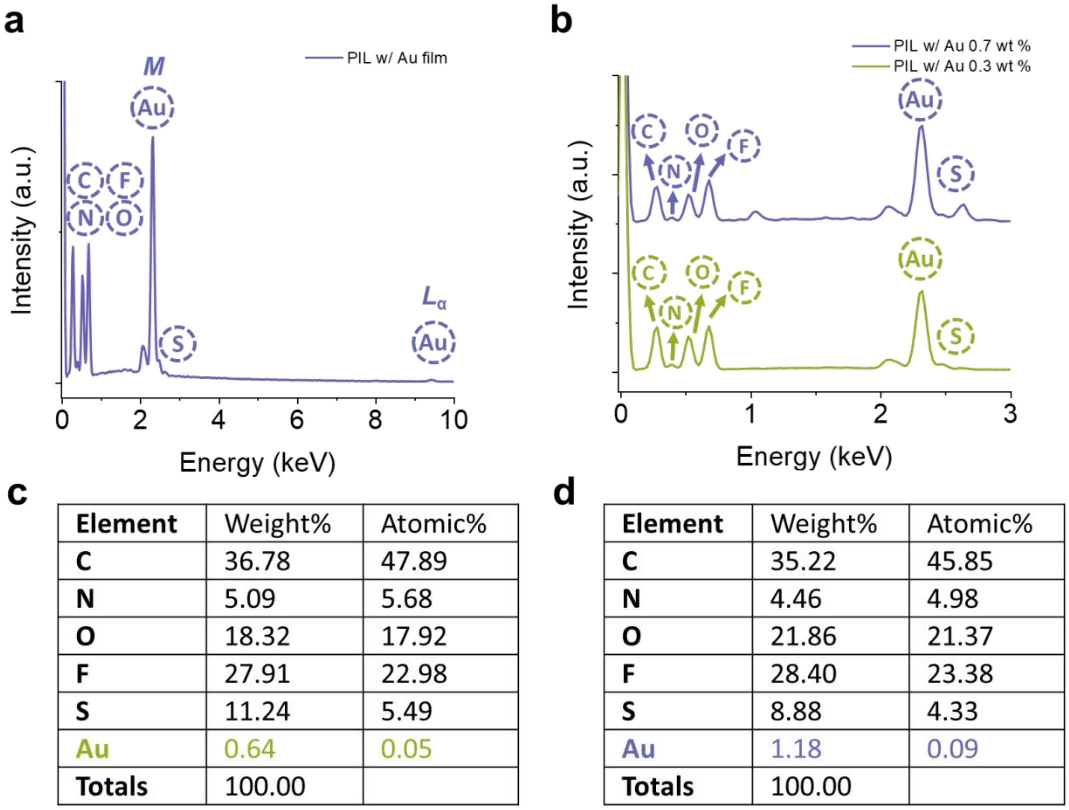

c

Element	Weight%	Atomic%
**C**	36.78	47.89
**N**	5.09	5.68
**O**	18.32	17.92
**F**	27.91	22.98
**S**	11.24	5.49
**Au**	0.64	0.05
**Totals**	100.00	

d

Element	Weight%	Atomic%
**C**	35.22	45.85
**N**	4.46	4.98
**O**	21.86	21.37
**F**	28.40	23.38
**S**	8.88	4.33
**Au**	1.18	0.09
**Totals**	100.00	

Figure S4. (a) EDS whole spectrum of a PIL w/ Au film. (b) EDS spectra of PIL w/ Au films with 0.03 and 0.07 wt % of gold contents. Elemental ratios of PIL w/ Au films with (c) 0.03 and (d) 0.07 wt % of gold contents.

a

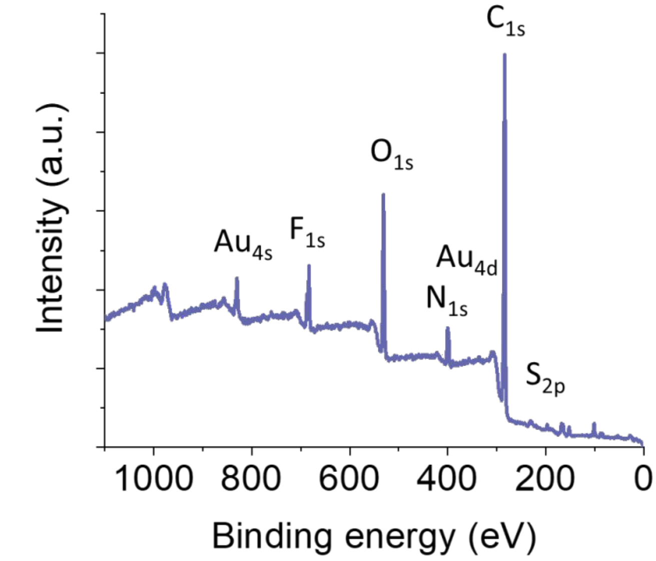

b

Element	Atomic%
**C $_{1s}$**	75.05
**N $_{1s}$**	5.88
**O $_{1s}$**	12.15
**F $_{1s}$**	3.96
**S $_{2p}$**	2.92
**Au $_{4d}$**	0.04
**Total**	100.00

Figure S5. (a) XPS spectrum and (b) elements ratios of PIL w/ Au films.

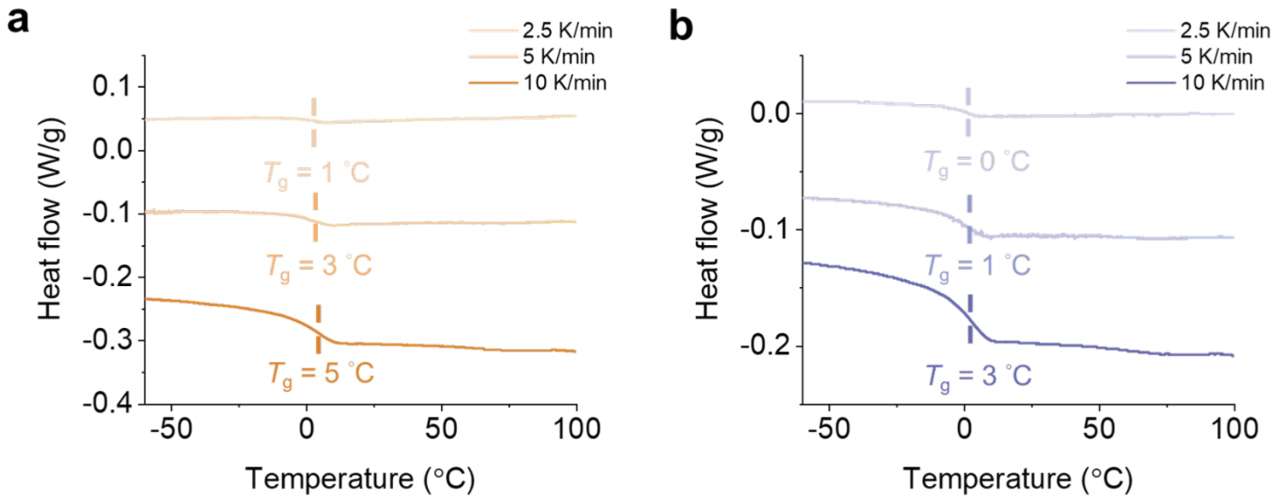

Figure S6. DSC curves of the (a) pristine PIL and (b) PIL w/ Au films under different scanning rates.

Table S1. Summarized table of glass transition temperatures (T_g) of the pristine PIL and PIL w/ Au films under different scanning rates

$T_{g,\ DSC}$ (°C)	2.5 K/min	5 K/min	10 K/min
**PIL**	1	3	5
**PIL w/ Au**	0	1	3

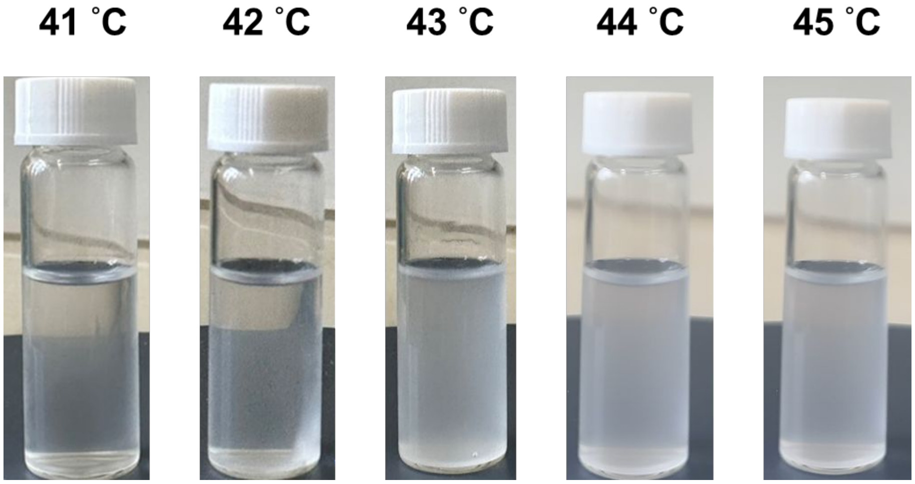

Figure S7. Images of the aqueous solution of 1 wt % PDMAEMA under different temperatures.

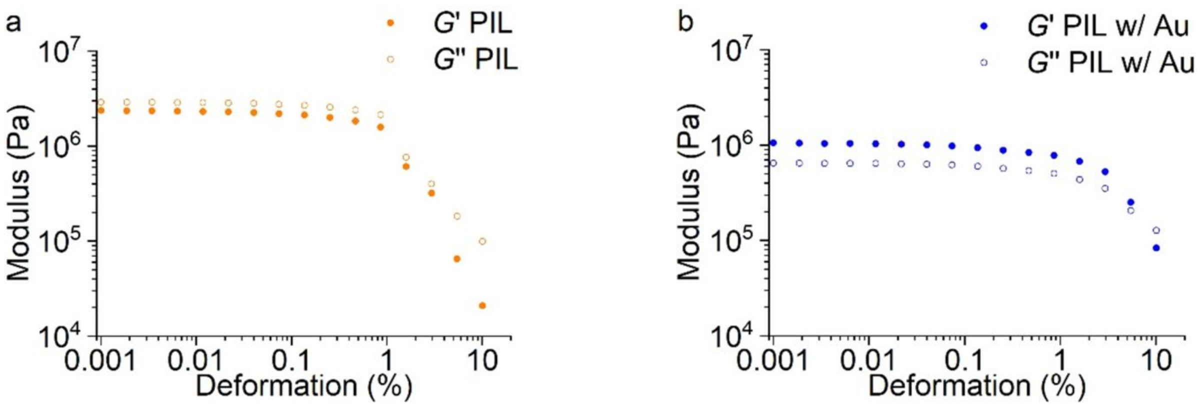

Figure S8. DMA measurements of (a) pristine PIL and (b) PIL w/ Au films displaying storage moduli (G') and loss moduli (G'') in amplitude sweep (ω = 10 rad/s, 25 °C).

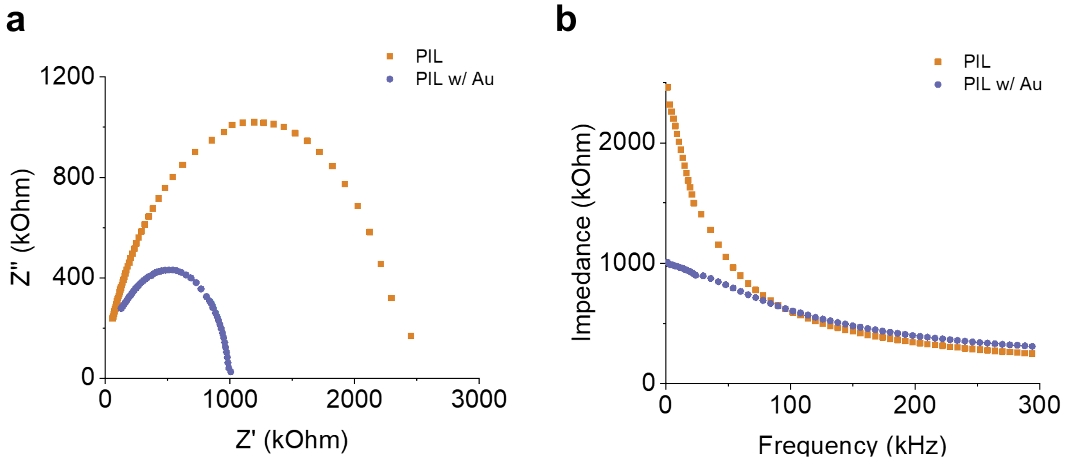

Figure S9. (a) Nyquist plots and (b) Bode plots of the pristine PIL and PIL w/ Au films.

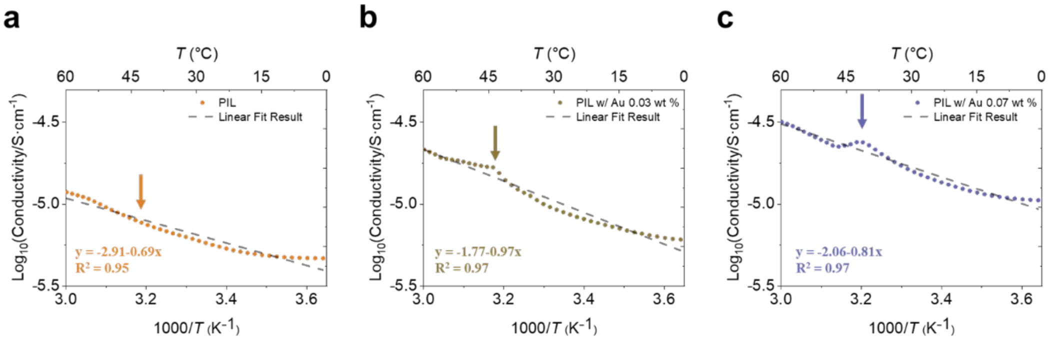

Figure S10. Temperature-dependent conductivity plots and linear fit result of the (a) pristine PIL, (b) PIL w/ Au films with 0.03 wt % of gold content, and (c) PIL w/ Au films with 0.07 wt % of gold content.

Table S2. Summarized table of the conductivity changes of the pristine PIL, PIL w/ Au films with 0.03 and 0.07 wt % of gold content

**Conductivities @40 °C**	**PIL**	**PIL w/ 0.03 wt %**	**PIL w/ 0.07 wt %**
**Fit Results**	7.40E-6	1.41E-5	2.40E-5
**Measured Results**	7.30E-6	1.32E-5	2.19E-5
$\Delta\sigma_{Fit\text{-}Measured}/\sigma_{Fit}$ **(%)**	1.37	6.67	8.80

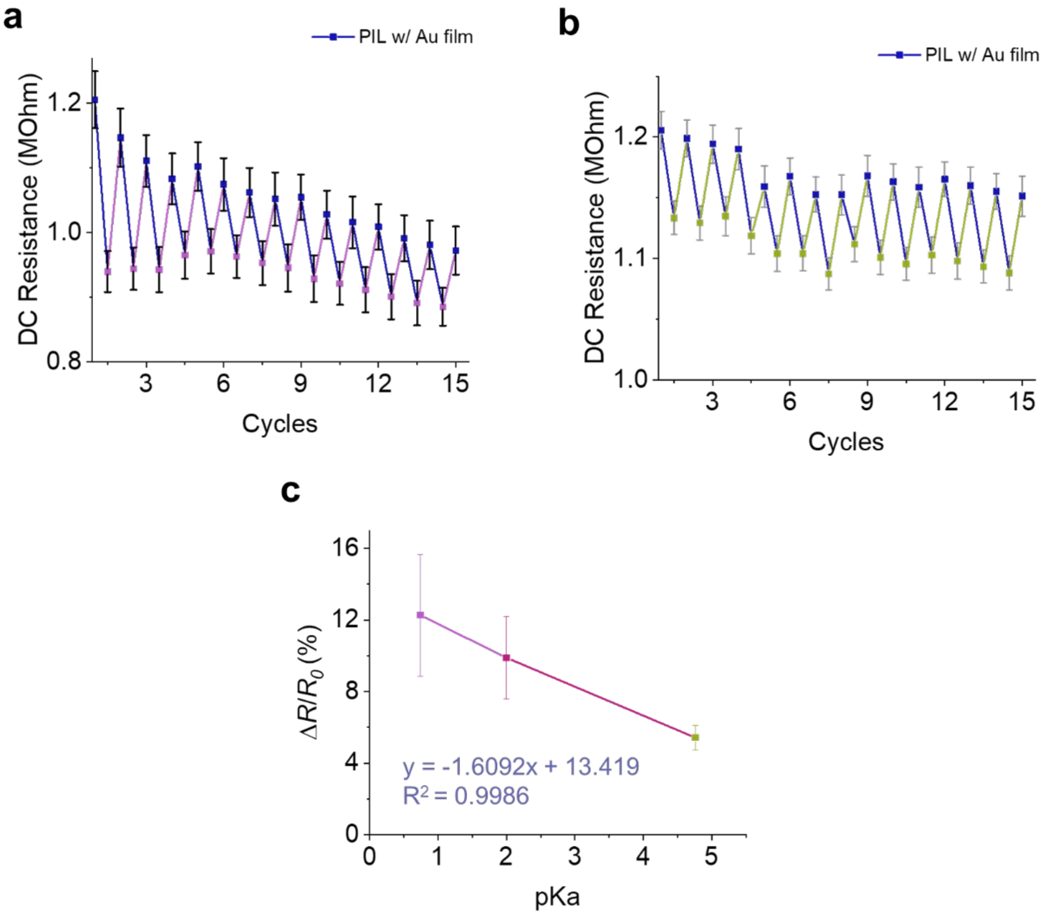

Figure S11. DC resistance changes of the PIL w/ Au films with 0.07 wt % of gold content under alkaline (ammonium hydroxide) and different acid vapors annealing: (a) trifluoroacetic acid (TFA) and (b) acetic acid. (c) DC resistance changing ratios under acid vapors with different pKa values.

Table S3. Summarized table of DC resistance changing ratios of the PIL w/ Au films with 0.07 wt % of gold content annealed under different acid vapors

**Acid**	**TFA** (pKa = 0.74)	**HCl** (pKa = 2)	**Acetic Acid** (pKa = 4.76)
**$\Delta R/R_0$ (%)**	12 ± 3	10 ± 2	5 ± 1

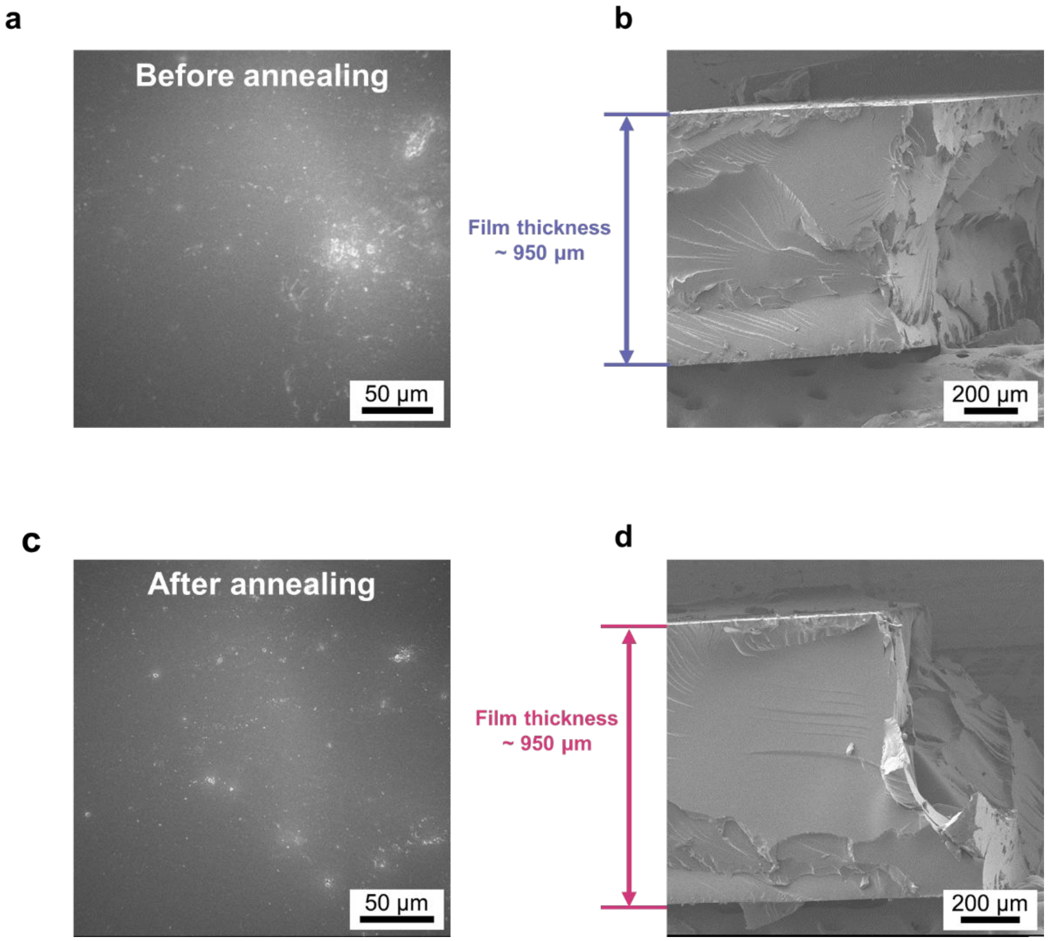

Figure S12. (a) Top-view and (b) cross-sectional view SEM images of PIL w/ Au films with 0.07 wt % of gold content before acid and alkaline annealing. (c) Top-view and (d) cross-sectional view SEM images of PIL w/ Au films with 0.07 wt % of gold content after acid and alkaline vapors annealing for over 50 cycles.

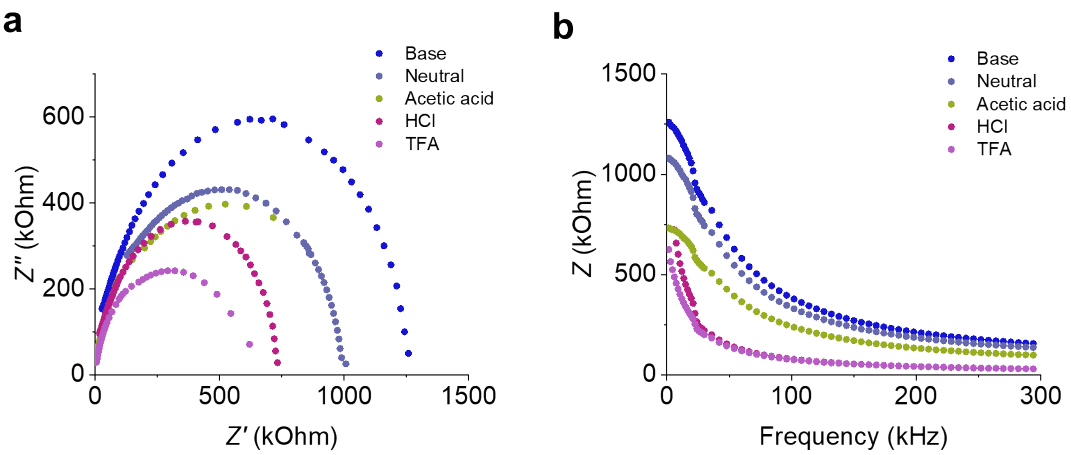

Figure S13. (a) Nyquist plots and (b) Bode plots of the PIL w/ Au films with 0.07 wt % of gold content under different pH value environments.
